# Use of Incident Command System for Disaster Preparedness: A Model for an Emergency Department COVID-19 Response

**DOI:** 10.1017/dmp.2020.210

**Published:** 2020-06-24

**Authors:** Andra Farcas, Justine Ko, Jennifer Chan, Sanjeev Malik, Lisa Nono, George Chiampas

**Affiliations:** McGaw Medical Center of Northwestern University, Chicago, Illinois; Northwestern Medicine Feinberg School of Medicine, Chicago, Illinois; Northwestern Medicine, Chicago, Illinois

**Keywords:** communication, coordination, COVID-19, disaster preparedness and response, incident command system

## Abstract

The COVID-19 pandemic has placed unprecedented demands on health systems, where hospitals have become overwhelmed with patients amidst limited resources. Disaster response and resource allocation during such crises present multiple challenges. A breakdown in communication and organization can lead to unnecessary disruptions and adverse events. The Federal Emergency Management Agency (FEMA) promotes the use of an incident command system (ICS) model during large-scale disasters, and we hope that an institutional disaster plan and ICS will help to mitigate these lapses. In this article, we describe the alignment of an emergency department (ED) specific Forward Command structure with the hospital ICS and address the challenges specific to the ED. Key components of this ICS include a hospital-wide incident command or Joint Operations Center (JOC) and an ED Forward Command. This type of structure leads to a shared mental model with division of responsibilities that allows institutional adaptations to changing environments and maintenance of specific roles for optimal coordination and communication. We present this as a model that can be applied to other hospital EDs around the country to help structure the response to the COVID-19 pandemic while remaining generalizable to other disaster situations.

Significant advances have been made in the realm of disaster preparedness over the past few decades. Models and systems, such as the United States National Incident Management System (NIMS), have been created to address challenges relating to communication, resource use, and coordination of efforts.^1-3^

Valuable lessons were learned from notable past disaster scenarios, such as Hurricane Katrina and the Boston Marathon bombings. In the former, communication was problematic as both cellular towers and landlines stopped working; hospitals switched to using ham radios and walkie-talkies as a result.^4^ During the Boston Marathon bombings, communication also became a problem when the cellular towers were shut down, as the primary interdepartmental method of communication was by means of cell phones.^5^ This resulted in delays in treatment and redundant trips and actions. Through these examples, breakdowns in communication were made evident. Uncertainty related to command structures, roles, and relationships led to confusion and limited a coordinated response.^6^

The global community, specifically hospital-based responses, are currently facing unprecedented challenges responding to the outbreak of the novel severe acute respiratory syndrome coronavirus-2 (SARS CoV-2) causing coronavirus disease 2019 (COVID-19). Hospitals across the world are evaluating and caring for rising numbers of infected patients, despite limited resources and increasing community panic. Prior efforts with multi-agency incident command systems (ICS) have proven effective for mass gatherings, such as the Chicago Marathon, and similar concepts can be translated to the current disaster state. Similarly, ICS principles have been used to organize responses to other major disasters and disease outbreaks. In more recent years, responses for disease outbreaks, such as the US response to Ebola and Zika, have required coordinated efforts through an ICS.^7^ Additionally, during the outbreak of SARS in Taiwan and the initial H1N1 influenza outbreak in Mexico, an ICS structure was adopted by respective health authorities to help organize responses.^8,9^ In the current disaster, the emergency department (ED) serves at the frontlines and, therefore, has a vital role in preparedness and response. It follows that an ICS structure adapted to respond to the COVID-19 pandemic needs to include an ED component to be maximally efficient.

Using guidelines from the Federal Emergency Management Agency (FEMA), we believe that a unified command approach within the ED during this unprecedented time can alleviate tasks and help coordinate a joint effort among hospital stakeholders.^10^ In this article, we specifically describe the ICS at our institution during the COVID-19 pandemic, focusing on the unique aspect of the ED serving as the Forward Command, which FEMA defines as the location nearest to the affected area used to direct activities and coordinate field teams.^11^ We believe that similar command structures and unified commands can assist hospitals in managing this growing pandemic and other analogous disasters.

## FEMA AND US FEDERAL GUIDELINES

Under the Department of Homeland Security, FEMA developed NIMS so that communities could create a “common, interoperable approach to sharing resources, coordinating and managing incidents, and communicating information.”^10^ This system was first implemented in 2004 in the aftermath of the 9/11 terrorist attacks. NIMS focuses on 3 pillars for the foundation for incident management: resource management, coordination and command, and communication and information management, while it is also guided by flexibility, standardization, and unity of effort.^10^

The notion of the ICS resulted from the development of NIMS and was defined as a “standardized approach to the command, control, and coordination of on-scene incident management that provides a common hierarchy within which personnel from multiple organizations can be effective.”^10^ It centers around having a predetermined chain of command that allows for a unified approach with a clear line of authority. It focuses on communication, especially interagency, and facilitates multiple agencies working together while allowing each agency to maintain their own authority.^10^ At its core, it is designed to alleviate “confusion regarding authorities and responsibilities” and reduce “resource shortages and misdirection of existing resources.”^12^ ICS also centers around the concept of a unified approach, so there is no single commander in the system but instead a unified set of objectives. This allows for full cooperation between various stakeholders and a union of similar goals and expectations. Each agency is responsible for presenting and sharing its agency-specific information, such as resource availability, limitations, and conflicts.^10^ These are crucial concepts in disaster environments, where an organized and coordinated response can minimize confusion while maintaining efficiency. The COVID-19 pandemic is an example of a complex unprecedented disaster and public health crisis requiring the efficient coordination of interdependent agencies with various activities.

The federal government requires the use of NIMS and ICS to structure a response to disaster scenarios.^13^ The ICS structure is used at all levels of a disaster response, from single departments to entire hospitals and, on a broader scale, at the city, state, and federal levels. These individual ICS structures integrate in a clearly defined manner, which is critical for coordinating an efficient response in a disaster situation. It is also important to ensure that implementing the ICS structure enhances response activities rather than encumbers them.^14^ We have, therefore, similarly adapted these principles to structure and enhance our hospital’s response to the current COVID-19 pandemic.

## SHARED MENTAL MODEL

During a disaster, decisions are often complex and fluid. They involve multiple stakeholders at various levels of command and authority with a wide array of backgrounds and experiences throughout different agencies. Decision-making in a disaster situation needs to be dynamic and distributed across different agencies that share common goals.^1^ It can often be difficult to unify everyone’s thoughts and objectives, which can have consequences on the efficiency of the disaster response. Therefore, it is important to have a shared mental model (SMM) to enhance teamwork, and a lack thereof can be an obstacle to interagency coordination. SMMs provide information to all of the team members regarding individual responsibilities. The model facilitates synergy between various parts of the team when a task needs to be achieved by interdependent work.^15^ Stout et al. showed that high-quality planning led to the development of a better SMM and performance in a teamwork scenario.^15^ During an inter-agency response to a railway accident in the United Kingdom, a poorly distributed SMM contributed to difficulty in coordination.^1^

We believe that the planning involved with setting up the proposed ICS structure and the structure itself helps encourage an SMM in disaster situations where coordination among various team members may otherwise be a point of weakness. During the COVID-19 pandemic, our SMM encompasses the judicious use of resources, such as personal protective equipment (PPE), isolation of the infected population, and protection of staff. This SMM enables hospital departments to work together during a surge to limit nonemergent visits and to focus on protocols to ensure staff protection. It is also important that there is an SMM at each level of the hospital ICS. At the ED level, it is critical that all parties share an SMM to design our ICS structure.

## ED FORWARD COMMAND

At the heart of our ICS model is the ED Forward Command. While the concept of a forward command, or a physical space nearest to the affected area where representatives of different agencies gather to coordinate and direct activities, is commonly used in other ICS structures, the concept of the ED serving as Forward Command in a hospital ICS is a fairly unique one.^11^ In designing this concept, we looked to the Chicago Model, a comprehensive disaster preparedness model for large mass gatherings, which uses an on-site Forward Command.^2,16^ This model has been used to coordinate the Chicago Marathon, one of the world’s largest marathons with over 45,000 annual participants. Although a large mass gathering event does not translate directly to an active large scale disaster response, the Chicago Model does represent a robust approach to establishing a framework to best respond to a complex large scale event.^2^

In the midst of a pandemic, the forward command concept can be easily translated to the ED, which is at the frontlines of this COVID-19 crisis. EDs around the country are the front doors through which COVID-19 patients enter into the hospitals and, along with intensive care units and general medicine floors, are the areas of the hospital most involved with the response to this pandemic.^17^ Because emergency staff are the first to interact with and treat affected patients, as well as are in direct contact with first responders, they are able to see the day-to-day changes in the course of the pandemic and understand which response strategies are efficient and which need to be changed. Similar to agencies gathering in the Forward Command in the Chicago Model, representatives from various areas of focus come together in the ED. This allows for real-time updates and an organized approach to solving problems from those experiencing them firsthand. It, therefore, follows that the ED is the department best suited operationally and physically to serve as the Forward Command.

An ED’s typical leadership structure can serve as the starting point for a forward command structure, which is how it was formed in our ICS. Instead of the external agencies involved in a traditional interagency/interdepartmental model, an ICS structure at the ED level is composed of ED leadership split into various areas of focus that function much like agencies do in the traditional ICS structure. Although existing ED leadership roles are a good organizational starting point, it is critical to adapt this to the needs of a particular incident or disaster. While a common academic ED model may not apply to a more clinically oriented disaster, items such as effects on research, educational mission, trainee safety, and wellness are unique aspects that warrant dedicated focus.

## INCIDENT COMMAND STRUCTURE ROLES

**FIGURE 1:**
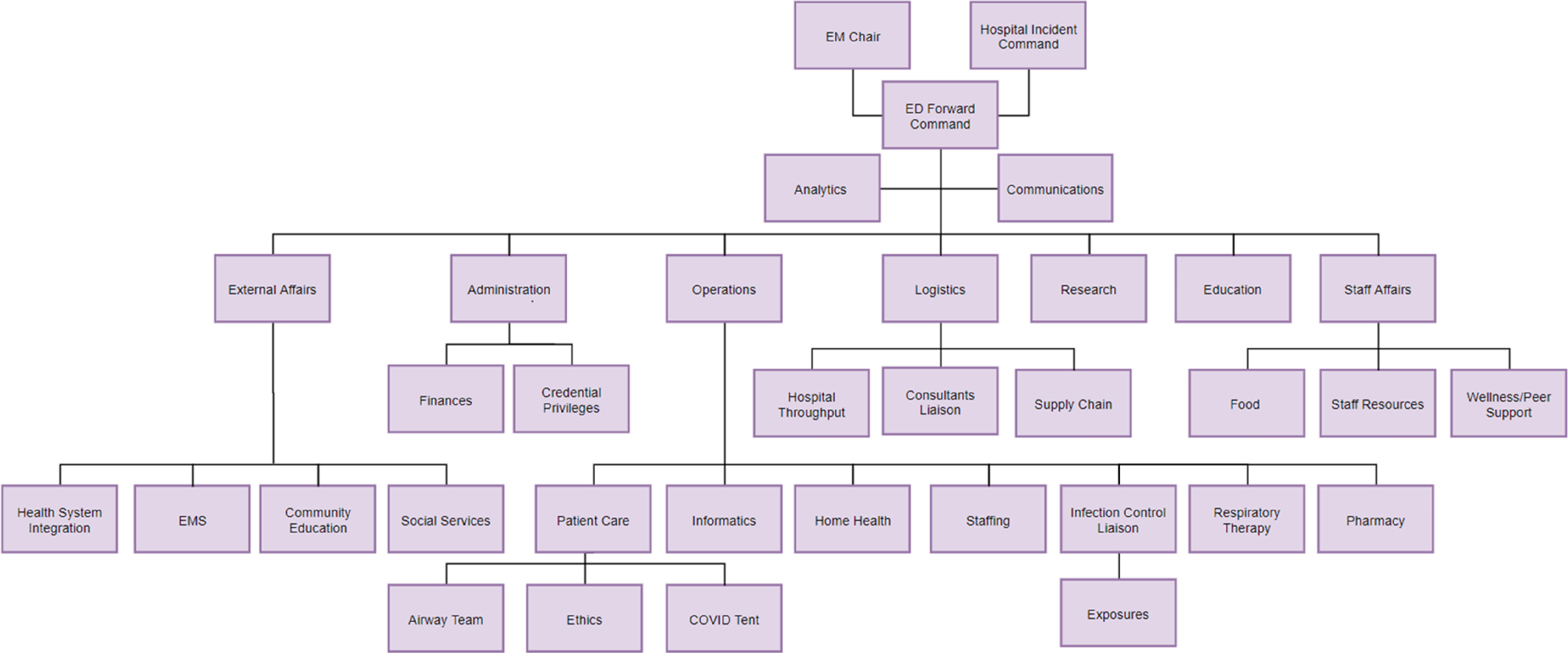
Emergency Department Incident Command System Structure

The ICS model’s unified command theory allows for the burden of response to be widely dispersed but interconnected with a common goal. It helps avoid overwhelming a single individual or department and instead spreads the unprecedented increase in workload across a larger group of stakeholders. Our ICS structure and involved areas of focus during the COVID-19 pandemic or other similar disasters can be seen in Figure 1. In our top-down model, the hospital Incident Command serves as the Joint Operations Center (JOC) and is the overall decision-maker for the incident. The emergency department falls under the Operations section chief and serves as Forward Command, managing the interdepartmental work of multiple areas of focus. Each of these areas has their own set of roles and responsibilities. A manager or representative is assigned for each area and serves as the liaison between their staff and the Forward Command team.

### Communications

This area is responsible for relaying ED-specific information to hospital media relations, hospital leadership, and ED personnel. It is also responsible for communicating externally with the approval of the JOC. It is tasked with creating a daily situation report that provides a composite timely overview of both the system-wide and ED-focused responses and incorporates ICS structural updates. Having a single team managing communication helps to limit misinformation to personnel and the public, which is especially important in a situation where clear and concise communication is vital for operations and mitigating staff and public panic.

### External Affairs

External Affairs is the area that oversees emergency medical services, community education, social services, and health system integration. It interacts with public/private partnerships, outside hospitals and care providers, as well as with local government, to provide community resources. For example, it engages with community partners to tackle the challenges that COVID-19 poses to the homeless population and coordinates with city-wide actors who are establishing a large field hospital for COVID-19 patients.

### Administration

Administration leads all financial and necessary administrative duties related to the ED and the current crisis. Examples include establishing policies for furloughed workers and expedited credentialing processes to allow surge staffing from the labor pool if necessary.

### Operations

Operations oversees all ED clinical procedures, patient care, and personnel across the department. One of the main roles during the pandemic is patient care, which includes innovative ways to prepare for anticipated patient surges. Operations must adapt to the challenges of the particular incident. In our department, a temporary COVID-19 unit (“COVID tent” in Figure 1) is set up outside of but adjacent to the ED to accommodate an influx of patients who require testing but may not need hospitalization. Additionally, the risk of health-care worker exposures experienced in other parts of the world prompted a change to typical airway management in the ED: a dedicated airway support team was created, which helps to ensure intubations are carried out in the safest manner possible. Operations is also responsible for staffing the department, which has required significant changes to adapt to a new patient population. Resource allocation and ethics also fall within this domain. The high transmission rates for COVID-19 has created unique circumstances and ethical dilemmas during patient care, such as early intubations and limiting family visitations. Specifically pertinent to the current COVD-19 pandemic, the Operations team also serves as the liaison with the infection control department and helps manage personnel exposures to the virus.

### Logistics

Logistics is responsible for department strategy and ensuring all processes and protocols involving resources and personnel are implemented. This includes overseeing the supply chain, relationships with consultants, and department throughput. Crucial to the current pandemic, Logistics is responsible for ensuring adequate PPE is available for personnel, as well as other vital supplies for patient care. Additionally, in our hospital, creative innovations have been set in place to help minimize potential exposures to consultants. For example, we have been implementing telemedicine practices by means of phone and video interviews when in-person consultation is not crucial. Logistics also oversees ED throughput and has coordinated ways to improve this through actions, such as delaying elective surgical cases to make inpatient beds available.

### Research

Research is tasked with keeping abreast of new developments and discoveries. This is especially important in the current pandemic, as COVID-19 is a novel virus and new information is surfacing on a daily basis. The Research team serves as a conduit for sharing the broader research efforts within the hospital, including the development of novel treatments. It also works to develop protocols that may lead to new practices while navigating potentially difficult ethical pathways. The Research team is also faced with new challenges, especially with patient enrollment, and has needed to adapt to changes with patient interactions.

### Education

Education’s role is to consolidate all digital, written, and academic material that is pertinent to the current pandemic. It is tasked with disseminating this information to all ED stakeholders to ensure consistent care across various individuals. In a teaching hospital, Education is also responsible for ensuring the continuing education of trainees in a safe manner. The COVID-19 pandemic creates some unique challenges to the typical academic teaching model and educational curriculum. Enhancements to videoconferencing and asynchronous learning have been created to maintain an adequate trainee environment.

### Staff Affairs

Staff Affairs covers all entities that relate to supporting the individual needs of staff members. This may include coordination of support resources from the community and food for personnel. Importantly, it is also responsible for addressing wellness and peer support by monitoring staff’s physical and emotional health during the crisis. It aids as needed with interventions, such as securing meals, involving chaplain services, and identifying measures for physical, familial, and psychological decompression and mitigation. Other wellness initiatives include optional debriefing conference calls, as well as weekly emails with wellness-based reads and uplifting news.

In our department, these roles are filled by ED attending physicians, administrators, nursing leadership, and a data analyst in management and leadership roles. Resident physicians, nurses, and other allied health workers work under their management. In a disaster, clear and decisive structure is critical to management, and individuals are selected who can not only best manage teams and tasks to meet the goals of the particular role but also embrace the shared mental model to influence the mission. Some roles coincide well with a leader’s prior base of expertise, such as the Medical Director and an ED Nurse Manager leading Operations or Vice Chair of Research leading Research. However, other roles are assigned to support the needs of the incident. Staff Affairs is a role that requires unique communication and collaboration skills along with extensive community relationships. In this response, this role is co-led by the Vice Chair of Academics and an ED Nurse Manager with this experience. External Affairs is headed by an attending with medical directorship experience for events outside the ED. The Administration role is led by the Department Administrator, while Analytics is provided by the department’s Data Analyst.

As described, each of these stakeholders has an area of focus for which they can offer their expertise and relevant points of contact. This enables efficient and goal-directed responses. For instance, the COVID-19 pandemic caused a unique challenge with safely performing intubations and other aerosolizing procedures. To conserve vital PPE and limit unnecessary provider exposure, multiple areas of focus worked together to create a protocol outlining the necessary equipment, personnel, and procedures with input from various stakeholders. Members from Operations for personnel and patient care, Logistics for supplies, Education for trainee education, and Communications for dissemination of protocols all collaborated and rapidly developed a solution to a new problem. This example highlights the importance of the ICS structure and Forward Command on a departmental level.

## COMMUNICATION METHODS

Particular emphasis was placed on communication methods within our department. This includes communication and leadership within a specific area of focus so that all team members are united in their actions. More importantly, however, it involves sharing ideas and adaptability among the different areas of focus, as well as communication with personnel. As the situation within the current COVID-19 climate is ever-changing, frequent precise communication is crucial. Stout et al. reported that teams that used more efficient communication methods performed better than teams that did not.^15^

As learned from the Boston Marathon bombings and Hurricane Katrina, having backup systems of communication in place is crucial.^4,5^ While not as directly applicable to a viral pandemic given the anticipated course does not involve loss of phone towers, these lessons nonetheless emphasize the importance of contingency plans.

The primary method of communication within our ICS structure involves a daily conference call with ICS managers. There is also an editable, Web-based document that is updated daily and accessible by all stakeholders. There are radios for backup if these 2 forms of communication were to fail.

The primary method of communication outward to personnel is by means of near-daily email updates from Communications. These mainly address protocols and practices, as well as new research and pertinent educational materials. They are organized into main ICS-based categories (eg, Operations, Logistics, Staff Affairs, etc.). Personnel can also opt in to access updates in the virtual space by means of a Web-based conference call led by ED Forward Command that takes place 2 d/wk.

## LIMITATIONS

This model is not without limitations. Primarily, this is 1 hospital’s newly developed model for responding to a quickly-changing disaster situation. Although there is constant feedback and improvement internally, there is not as of yet any data to allow for an objective evaluation of such an approach. Further objective analysis of the efficiency and usefulness of this model is needed.

Furthermore, this model was developed in a large academic hospital in an urban environment and is, therefore, most applicable to similar EDs. It may be challenging for smaller hospitals and EDs to implement, as they may not necessarily have the staff or capabilities. Additionally, some hospitals may not have a need to address educational or research challenges. However, it is possible that the areas of focus can be combined for a more compact approach to the model, requiring fewer stakeholders.

Another evident limitation is that key leaders of the ICS also serve as staffing personnel. This may be challenging in the efficiency and timing of key communications but will be addressed in the after action.

## CONCLUSIONS

While disaster preparedness has developed significantly, the COVID-19 pandemic has led to novel and unprecedented demands on the health-care system. Using principles of NIMS and lessons learned from prior disasters and the successful implementation of similar systems in mass gatherings, we have developed a top-down approach to setting up an ICS in our hospital with the ED as the Forward Command. We propose that, in the current COVID-19 pandemic, this model can help improve communication, resource use, staff and patient safety, and maintenance of roles. We offer this model as one that can be generalizable to other sites and events but emphasize the need for adaptability to customize to the unique needs of both the hospital and the incident.
